# NEUROD1: transcriptional and epigenetic regulator of human and mouse neuronal and endocrine cell lineage programs

**DOI:** 10.3389/fcell.2024.1435546

**Published:** 2024-07-22

**Authors:** Gabriela Pavlinkova, Ondrej Smolik

**Affiliations:** Laboratory of Molecular Pathogenetics, Institute of Biotechnology Czech Academy of Sciences, Vestec, Czechia

**Keywords:** cell reprogramming, neurogenesis, cell therapy, pancreas, bHLH transcription factor

## Abstract

Transcription factors belonging to the basic helix-loop-helix (bHLH) family are key regulators of cell fate specification and differentiation during development. Their dysregulation is implicated not only in developmental abnormalities but also in various adult diseases and cancers. Recently, the abilities of bHLH factors have been exploited in reprogramming strategies for cell replacement therapy. One such factor is NEUROD1, which has been associated with the reprogramming of the epigenetic landscape and potentially possessing pioneer factor abilities, initiating neuronal developmental programs, and enforcing pancreatic endocrine differentiation. The review aims to consolidate current knowledge on NEUROD1’s multifaceted roles and mechanistic pathways in human and mouse cell differentiation and reprogramming, exploring NEUROD1 roles in guiding the development and reprogramming of neuroendocrine cell lineages. The review focuses on NEUROD1’s molecular mechanisms, its interactions with other transcription factors, its role as a pioneer factor in chromatin remodeling, and its potential in cell reprogramming. We also show a differential potential of NEUROD1 in differentiation of neurons and pancreatic endocrine cells, highlighting its therapeutic potential and the necessity for further research to fully understand and utilize its capabilities.

## 1 Introduction

The differentiation of specialized cells during development involves complex molecular and cellular processes. Comprehending these differentiation processes is key to understanding cellular function in both normal and diseased states. Moreover, it helps to devise novel strategies to manipulate and reprogram cells for therapeutic applications. Transcription factors have instructive role for the generation of functionally differentiated cells from pluripotent progenitors via sequential fate temporal and spatial restriction steps. The combinations of transcription factors define differentiation cell lineages, differentiation stages, as well as lineage reprogramming and developmental transdifferentiation.

NEUROD1 (Neurogenic differentiation factor 1) is a transcription factor that plays crucial roles in both the nervous system and the pancreas. Initially characterized for its ability to convert nonneural ectodermal cells into neurons in *Xenopus* embryos (Lee et al., 1995). Nervous system development is a multi-step process that generates a multitude of cell types. Studies in all major model organisms, *Caenorhabditis elegans*, *Drosophila melanogaster*, and mice demonstrated that NEUROD1 and its homologs are critical regulators of neuronal progenitor cell differentiation and neuronal specification (Bertrand et al., 2002; Hallam et al., 2000; Gao et al., 2009; Liu et al., 2000a; Miyata et al., 1999). Initially characterized for its involvement in neuronal differentiation, NEUROD1 has since been identified as a key regulator in pancreatic endocrine development and function (Gu et al., 2010; Romer et al., 2019; Bohuslavova et al., 2023; Naya et al., 1997). This review aims to provide an integrated perspective on diverse roles of NEUROD1, focusing on its contributions to neuroendocrine development, and associated disorders in humans and mouse models. Our review is organized into four thematic areas: a) NEUROD1 in neuronal development; b) NEUROD1 in differentiation of pancreatic endocrine cells; c) therapeutic potential of NEUROD1 as a reprogramming factor; and d) the role of NEUROD1 in neuroendocrine tumorigenesis. We integrate the current state of knowledge into a broader context of biological function and clinical applications.

### 1.1 bHLH transcription factors

The basic helix-loop-helix (bHLH) superfamily of transcription factors is characterized by two distinct domains, the basic domain at the amino-terminal end, which binds the transcription factor to DNA to a short CANNTG motif known as Ephrussi-Box (E-box), and the HLH domain at the carboxy-terminal end, which facilitates interactions with other protein subunits to form homo- and hetero-dimeric complexes (Jones, 2004). The functional and evolutionary classification of animal bHLH proteins into six groups, A through F, is based on criteria such as E-box binding, sequence comparisons, tissue distribution, phylogenetic relationships, and the presence/absence of additional domains (Atchley and Fitch, 1997; Ledent and Vervoort, 2001). Group B proteins are found in plants, yeast, and animals, whereas the other groups (A, C, D, E, and F) are found only in animals. Majority of bHLH proteins are members of A or B groups, characterized by their ability to bind to core E-box consensus sequences through a distinctive pattern of amino acids at positions 5, 8, and 13, known as the 5-8-13 E-box configuration. Group A includes several tissue-specific bHLH proteins, such as MyoD, Achaete-Scute, Twist, and also ubiquitously distributed E12/Daughterless-type bHLH proteins. Group C represents a separate lineage derived from Group B, distinguished by its lack of a consistent amino acid configuration at sites 5, 8, 13 and its possession of a pair of PAS domains, which are signaling modules (Crews and Fan, 1999). Groups D and F represent proteins diverged at the basic region of the bHLH domain. Group D proteins do not bind DNA; instead, they act as dominant-negative regulators by forming dimers with other bHLH proteins, thereby inhibiting their DNA binding activity. Group F comprises the so-called COE proteins (collier/olfactory-1/early B-cell factor), which contain a unique COE domain important for both dimerization and DNA binding. The HLH motifs of this group are highly divergent from those found in other bHLH proteins. Members of the bHLH superfamily regulate an abundance of biological processes and developmental pathways, such as cell lineage determination, differentiation, response to environment, cell cycle and proliferation (Murre, 2019).

BHLH factors bind DNA as either homodimers or heterodimers, with their partner selection critically influencing their binding properties, impacting their spatial and temporal regulatory function (see reviews (Murre, 2019; de Martin et al., 2021). Unlike other bHLH groups, group D factors lack a DNA-binding domain and form inactive heterodimers with other bHLH proteins, acting as negative regulators. Additionally, bHLH factors bind other cofactors for an effective repression or activation of transcription of their target genes. Particularly, different combinatorial interactions of bHLH and other transcription factors expressed in different locations and/or times influence the context-dependent bHLH specific target gene regulation. For example, the spatiotemporal control of neurogenesis and gliogenesis in the developing ventral spinal cord is performed in collaboration of bHLH factors OLIG2, NEUROG1-3, and ASCL1 with NKX2.2, and PAX6 (Sugimori et al., 2007). Different combinations of transcription factors have an ability to either inhibit or enhance the activity of the proneural bHLH NEUROGs and ASCL1. OLIG2 suppresses the neurogenic activity of NEUROG2 and enhances ASCL1-dependent oligodendrogenesis, whereas PAX6 or NKX2.2 interactions promote proneurogenic regulation of ASCL1 (Sugimori et al., 2007).

Additionally, bHLH factors interact with chromatin remodelers, histone modifiers, and enzymes regulating DNA methylation, altering the epigenome landscape. For example, the catalytic subunit BRG1 of the chromatin remodelling complex SWI/SNF interacts with proneural bHLH proteins such as NEUROG1 and NEUROD1 during neurogenesis (Seo et al., 2005). The lineage-specific bHLH transcription factors bind closed chromatin and recruit chromatin remodelers (Wapinski et al., 2013). The regulatory complexities of three key bHLH factors—ASCL1, a regulator of neural lineage differentiation; ASCL2, crucial for the development of lineages like trophectoderm, T-helper cells, and intestinal stem cells; and MYOD1, a master regulator of skeletal muscle differentiation—were explored in a study using embryonic stem cells engineered to ectopically express each bHLH factor. This study revealed that transgenic expression of these bHLH factors induced significant changes in histone modifications and enabled their binding previously inaccessible (closed) chromatin sites, underscoring their roles in chromatin remodeling and lineage-specific gene regulation (Casey et al., 2018). The ability to bind inaccessible chromatin is known as “pioneering,” and factors, termed as “pioneer factors”, can access their target genes in silent highly packed chromatin. Pioneer factors initiate the recruitment of other regulatory proteins and activate gene transcription to induce cell fate changes in development and cell reprogramming (Iwafuchi-Doi and Zaret, 2016).

### 1.2 NEUROD1 as a proneural bHLH factor in neurons

Proneural bHLH factors are important regulators in neuronal specification, differentiation, neural cell fate, and self-renewal (Imayoshi and Kageyama, 2014; Fritzsch et al., 2015; Baker and Brown, 2018; Dennis et al., 2019). Neural-specific bHLH factors are subdivided into the achaete-scute complex (AS-C) and atonal gene families based on their homology to *Drosophila* genes (Dennis et al., 2019). The AS-C family is represented by achaete-scute like bHLH gene family (*Ascl1-Ascl5*) in the mouse. The distantly related are *Nscl* family genes (*Nhlh1/Nscl1, Nhlh2/Nscl2*). The atonal family is represented by multiple bHLH genes, including members of the *Neurogenin* (*Neurog1*, *Neurog2*, *Neurog3*), *Atonal* (*Atoh1/Math1, Atoh7/Math5*), *Olig* (*Olig1, Olig2, Olig3, Bhlhe22/Bhlhb5*), and *Neurod* (*Neurod1, Neurod2/Ndrf, Neurod6/Math2, Neurod4/Math3*).

One of the earliest discovered proneural bHLH transcription factors is Neuronal differentiation 1 (NEUROD1), identified due to its ability to convert epidermal cell fate into neuronal cell fate (Lee et al., 1995). During embryonal development, NEUROD1 is expressed in all areas of the brain, spinal cord, peripheral ganglia, sense organs, and endocrine pancreas that expresses the bHLH factors, Neurogenins 1, 2, and/or 3 [NEUROG1, 2, and 3 (Sommer et al., 1996)]. Recent studies employing systematic or tissue-specific deletions of the *Neurod1* gene have revealed a consistent pattern of neurological abnormalities alongside a severe neonatal diabetes phenotype (Bohuslavova et al., 2021; Naya et al., 1997; Romer et al., 2019; Bohuslavova et al., 2023). *Neurod1* deletions disrupt neurogenesis within the central nervous system, impacting the differentiation of key neuronal populations, including cerebellar granule cells, dentate gyrus cells, and newborn neurons derived from neural stem cells in the adult hippocampus and olfactory bulb (Hevner et al., 2006; Miyata et al., 1999; Schwab et al., 2000; Liu et al., 2000a; Gao et al., 2009; Pan et al., 2009). Additionally, NEUROD1 is required for the survival and differentiation of newborn neurons in the adult hippocampus subgranular and subventricular zones, as well as their maturation and integration into the neuronal circuitry (Gao et al., 2009).

Despite the widespread expression of NEUROD1 in the peripheral nervous system of mice, substantial defects in neurogenesis have been only reported in the development of sensory neurons of vestibular and spiral ganglia in *Neurod1* gene deletion mutants (Liu M et al., 2000; Kim et al., 2001; Jahan et al., 2010; Macova et al., 2019; Filova et al., 2022; Pyott et al., 2024). NEUROD1 is essential for the initiation of neurogenesis, delamination of neuroblasts, and survival of early neurons in the inner ear (Filova et al., 2022). In contrast, in the olfactory epithelium, the elimination of NEUROD1 does not affect initial olfactory sensory neuron differentiation but it compromises the production and survival of mature neurons of the olfactory epithelium (Packard et al., 2011). In the retina of mice deficient in the *Neurod1* gene, the progressive degeneration of rod and cone photoreceptors occurs, and amacrine cell differentiation is delayed (Morrow et al., 1999; Pennesi et al., 2003). This limited effect of NEUROD1 elimination may be attributed to functional redundancy with other bHLH factors involved in neuronal differentiation, such as NEUROD2, NEUROD4, NSCL1/NHLH1, and NEUROG2 (Vermeiren et al., 2020).

Although NEUROD1 is an essential mediator of neuronal fate specification and neuronal differentiation, its direct targets during neurogenesis remain largely unknown. To uncover NEUROD1 targets and regulatory networks of neurogenesis, NEUROD1 was induced ectopically in pluripotent mouse ES cells (Pataskar et al., 2016). This study demonstrated that NEUROD1 overrides the pluripotent state and promotes neurogenesis by direct binding to promoters and enhancers of neuronal developmental genes. Identified target sites were tested by performing Chromatin Immunoprecipitation (ChIP) assay in the mouse E13.5 cortex. Both experiments established that NEUROD1 binds regulatory elements of neuronal developmental genes, including *Pou3f2, Insm1, Nhlh1, Atoh1*, *Neurod4*, or *Nhlh2* (Pataskar et al., 2016).

### 1.3 NEUROD1 role in the developing pancreas

The pancreas has two essential functions in the body: endocrine, which involves the production of hormones that regulate glucose metabolism, and exocrine, which involves the production of digestive enzymes (Karpińska and Czauderna, 2022). The endocrine component of the pancreas consists of the islets of Langerhans, small sparse spherical cell clusters. These clusters contain five hormone-producing cell types: α cells secreting glucagon, β cells secreting insulin, γ cells producing pancreatic polypeptide, δ cells secreting somatostatin, and ε cells producing ghrelin. Dysfunction of pancreatic endocrine cells, particularly the failure of β cells to produce insulin, results in *diabetes mellitus*. The incidence of diabetes is increasing worldwide, necessitating the development of innovative therapies to either compensate for decreased insulin levels or replace dysfunctional β cells. NEUROD1 plays a critical role in the functional development of the endocrine pancreas, as *Neurod1* gene deletions are postnatally lethal within a few days after birth due to severe diabetes (Bohuslavova et al., 2021; Naya et al., 1997; Romer et al., 2019; Bohuslavova et al., 2023). Mutations in the *NEUROD1* gene in humans are linked to type 2 diabetes (Malecki et al., 1999) and a subtype of maturity-onset diabetes of the young (MODY6) (Malecki et al., 1999; Rubio-Cabezas et al., 2010; Horikawa and Enya, 2019). In mice, NEUROD1 becomes detectable in the endocrine cells of the developing pancreas as early as E9.5 (Naya et al., 1997; Chu et al., 2001; Bohuslavova et al., 2021). While it eventually diminishes from most of the endocrine subtypes (Itkin-Ansari et al., 2005), NEUROD1 remains actively expressed postnatally in mature β cells, most notably to function as a crucial transcription factor ensuring β-cell maturity (Gu et al., 2010; Bohuslavova et al., 2023) and physiological response of insulin gene to glycemic levels (Naya, Stellrecht, and Tsai, 1995; Romer et al., 2019; Bohuslavova et al., 2021). The postnatally lethal phenotype of severe hyperglycemia was linked to massive decrease of β cells and insulin expression between E14.5 and E17.5 (Naya et al., 1997). The expression of other endocrine peptides in the *Neurod1*-deficient pancreas, namely, glucagon, somatostatin, and pancreatic polypeptide, was reduced as well. Correspondingly, significant changes in the molecular identities of endocrine β-, α-, and PP-cell subpopulations were identified in *Neurod1* deletion mutant (Bohuslavova et al., 2023). Histological analyses revealed that deletion of the *Neurod1* gene led to a significant reduction in β-cell mass and notable alterations in islet morphology. The endocrine-cell loss was at first attributed to apoptosis (Naya et al., 1997), and later to decreased proliferation rate (Romer et al., 2019; Bohuslavova et al., 2021), or to combination of both (Dudek et al., 2021). The loss of endocrine cells occurs in cases where the *Neurod1* gene is deleted during the embryonic developmental phase but not in mature β cells (Gu et al., 2010).

NEUROD1 is a vital part of a complex gene regulatory network driving pancreatic endocrine differentiation. The double-knockout *Nkx2.2*;*Neurod1* studies showed that these two transcription factors co-orchestrate the basal balance in endocrine cells specification (Chao et al., 2007; Mastracci et al., 2013). NEUROD1 reportedly favors β cell-fate, while overabundant NKX2.2 tips the scales more towards α-, PP-, and ε-cell-fate prior E15.5, until NEUROD1 prevails and triggers massive β-cell expansion (Chao et al., 2007). The study in differentiated human ESC-β cells with the disruption of the *NEUROD1* gene demonstrated reduced expression of essential β-cell transcription factors, including MAFA, NKX6-1, PDX1, INSM1, NKX2-2, ISL1, PAX6, and RFX6 (Romer et al., 2019). These data suggest that NEUROD1 is essential for regulating the β-cell transcription factor network in humans (Romer et al., 2019). Overall, this situates NEUROD1 as a potent factor, which is required for endocrine differentiation as well as for the function of specialized mature β cells.

### 1.4 NEUROD1-induced cell-reprogramming

**FIGURE 1:**
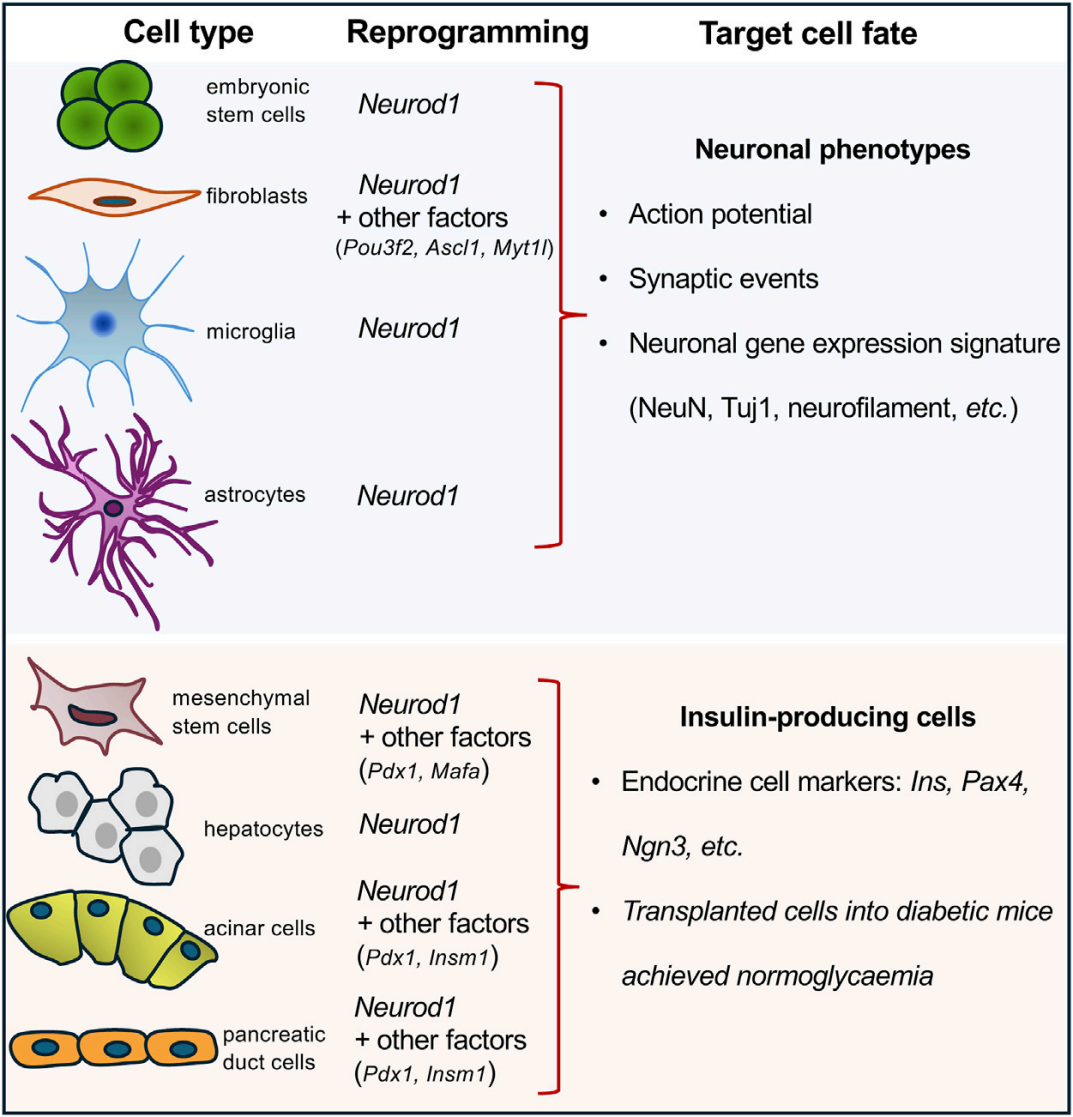
Overview of cell-reprogramming experiments with a delivery of *Neurod1*. Using *Neurod1* as a part of reprogramming cocktails or as a single factor in the reprogramming experiments of different cell types towards neuronal or β-cell-like phenotypes. See Table 1.

NEUROD1 binds regulatory elements of neuronal genes in closed heterochromatin and promotes epigenetic changes at its target sites to induce neuronal differentiation (Pataskar et al., 2016). Binding of NEUROD1 is associated with the loss of the repressive histone mark, trimethylation of lysine 27 on histone H3 (H3K27me3), a specific post-translational modification of the histone protein H3. This is accompanied by the gain of the active mark, acetylation of lysine 27 on histone H3 (H3K27ac) (Pataskar et al., 2016). This study demonstrated that NEUROD1 enhances the neurogenic potential of uncommitted cells, and that the induction of neuronal fate genes is maintained via epigenetic memory despite a transient NEUROD1 expression. Moreover, the deletion of the *Neurod1* gene in the developing pancreas was associated with significant changes in the endocrine cell epigenetic landscape, altering the H3K27me3 histone modification pattern in promoter regions (Bohuslavova et al., 2023). NEUROD1’s regulatory function in neurogenesis is further highlighted by its ability to bind target sites in heterochromatin, remodel chromatin, and initiate the conversion of heterochromatin to euchromatin, establishing its role as a pioneer factor (Pataskar et al., 2016). Together, the NEUROD1-induced changes in the epigenetic landscape at its target sites represent persistent changes and a sustained commitment of cells to NEUROD1-induced gene expression programs. This ability to drive cell-fate commitment makes NEUROD1 a compelling target for therapeutic cell programming. NEUROD1 has been utilized in reprogramming cocktails or as a single factor in experiments aimed at generating neuronal phenotypes or insulin-producing β cells (Figure 1). Key findings from various *in vivo* and *in vitro* reprogramming experiments utilizing NEUROD1 to induce transdifferentiation across different cell types, are summarized in Table 1.

**TABLE 1:** Overview of cell reprogramming studies involving *Neurod1*.

Starting cell type	Target cell fate	Reprogramming factor or factors	Delivery methods	Species and context	References
Primary hepatocytes	Endocrine cells	*Neurod1*	DNA via AAV	Mouse; *in vitro* culture	Yatoh et al. (2007)
H4IIE Rat Liver Cell Line	Beta-like cells	*Neurod1 or Neurod1+INS*	DNA via RV	Rat; *in vitro* culture	Ren et al. (2016)
Stromal cell lines	Endocrine islets	*Neurod1, Pdx1, and Ngn3*	DNA plasmid via lipofectamine	Human cell line; transplanted into the kidney parenchyma	Zhao et al. (2008)
Mesenchymal stem cells	Insulin-producing cells	*Neurod1, Pdx1, and Mafa*	DNA via AAV	Mouse; cells transplanted into mice with STZ-induced diabetes	Guo et al. (2012)
Pancreatic acinar cells and cell lines	Insulin-producing cells	*Neurod1, Pdx1, and Insm1*	DNA via AAV	Mouse; *in vitro* culture	Zhang et al. (2012)
Pancreatic duct cells	Insulin-producing cells	*Neurod1, Pdx1, and Insm1*	DNA via AAV	Human; *in vitro* culture	Zhang et al. (2010)
Reactive astrocytes	Neurons	*Neurod1*	DNA via RV	Mouse; *in vivo* the cortex of stab-injured or Alzheimer’s disease (AD) model mice	Guo et al. (2014)
Astrocytes	Neurons	*Neurod1*	DNA via RV	Human; *in vitro* culture	Guo et al. (2014)
Pluripotent mouse ES cells	Neurons	*Neurod1*	DNA via LV	Mouse; *in vitro* culture	Pataskar et al. (2016)
Embryo brain	Neurons	*Neurod1*	DNA electro-poration	Mouse; embryo brain	Pataskar et al. (2016)
Human fetal and postnatal fibroblasts	Neurons	*Neurod1 + Pou3f2, Ascl1, Myt1l*	DNA via LV	Human; *in vitro* culture	Pang et al. (2011)
Microglia	Induced neurons	*Neurod1*	DNA via LV	Mouse; *in vivo* mouse brain	Matsuda et al. (2019)
Mixed glial cell culture	Neurons	*Neurod1*	DNA via LV	Mouse; *in vitro* culture	Matsuda et al. (2019)
Astrocytes *in vivo*	Neurons	*Neurod1*	DNA via AAV	Mouse; *in vivo* the brain (cortex, striatum)	Brulet et al. (2017)
Müller glial cells in the retina	Retinal neurons	*Neurod1*	DNA via AAV	Mouse; *in vivo* the eye	Xu et al. (2023)

AAV, adeno-associated viral delivery; LV, lentiviral delivery; RV, retroviral delivery; STZ, streptozotocin.

For example, ectopic expression of NEUROD1, in combination with POU3F2, ASCL1, and MYT1L induced *in vitro* transdifferentiation of both human fetal and postnatal fibroblasts into functional induced neuronal cells (Pang et al., 2011). Similarly, NEUROD1, as a part of another reprogramming cocktail containing ASCL1, ISL1, BRN2, HB9, LHX3, MYTL1, and NEUROG2, reprogrammed mouse and human fibroblasts into motoneurons (Son et al., 2011). Additionally, non-neural cells can be reprogrammed into neuronal cells through the ectopic expression of NEUROD1 in combination with other factors using expression viral vectors, as demonstrated in various studies. For example, the combination of ASCL1 and NEUROD1 successfully reprogrammed spiral ganglion non-neuronal cells (Noda et al., 2018) and cochlear non-sensory epithelial cells into neuronal phenotypes (Nishimura et al., 2014). Furthermore, adipose-derived stem cells were reprogrammed into neurons using a combination of transcription factors including BRN2, ASCL1, BAM, and NEUROD1 (Petersen et al., 2015). Pathbreaking research demonstrated that NEUROD1 can act as a single reprogramming driver, which could induce *in vivo* conversion of reactive glial cells into functional neurons in the adult mouse cortex when infected with retrovirus encoding the *Neurod1* gene (Guo et al., 2014). During the last decade, ectopic expression of NEUROD1 using viral constructs carrying the *Neurod1* gene has been shown *in vivo* and *in vitro* experiments to promote neuronal fate and induce differentiation of neurons from astrocytes, retinal glial cells, and microglia (Brulet et al., 2017; Guo et al., 2014; Xu et al., 2023; Matsuda et al., 2019). However, recent studies have raised concerns about these findings, suggesting that some of the observed effects could be attributed to experimental artifacts, viral leakages, and insufficient lineage-tracing (Wang L. L. et al., 2021; Rao et al., 2021; Wang and Zhang, 2022). Nevertheless, there is ample evidence supporting the neurogenic activity of NEUROD1 in endogenous neural stem cells, glioblastoma and astrocyte cell cultures, and embryonic stem cells (Pataskar et al., 2016; Wang et al., 2021b; Guo et al., 2014). Altogether, these cell-reprogramming experiments suggest that NEUROD1 can initiate cell-fate changes and induce neurogenesis, underscoring the importance of timing and cell context for the ultimate outcome.

The development of cell therapy for insulin-dependent diabetes focuses on generating immuno-compatible insulin-producing cells capable of physiological glycemic regulation. Recent studies have explored reprogramming various cell types into insulin-producing endocrine cells by using viral expression constructs encoding the *Neurod1* gene in combination with other key regulatory factors. These reprogramming efforts have targeted different progenitor and somatic cell types: hepatocytes transfected with viral vectors encoding the *Neurod1* gene (Yatoh et al., 2007; Ren et al., 2016); mesenchymal stem cells reprogrammed with viral vectors encoding gene combinations of *Pdx1*, *Neurod1*, and *Ins* (Gerace et al., 2019) or *Pdx1, Neurod1,* and *Mafa* (Guo et al., 2012); marrow-derived stromal cell lines (Zhao et al., 2008; Li et al., 2017) targeted with viral vectors encoding *Pdx1, Neurod1,* and *Neurog3*; and both pancreatic acinar cells (Zhang et al., 2012) and pancreatic ductal cells (Zhang et al., 2010) were reprogrammed using viral vectors encoding *Pdx1*, *Neurod1*, and *Insm1* genes. Despite the variety of successful reprogramming protocols, the ability of NEUROD1 to induce the generation of endocrine insulin-producing cells is relatively limited compared to its more robust efficacy in promoting neuronal phenotypes. This limitation indicates that while NEUROD1 plays a significant role in neural reprogramming, its use for generating insulin-producing cells may require additional factors or conditions to achieve optimal results.

While the search for the optimal solution for patient-tailored gene therapy is an ongoing challenge, the molecular mechanism behind the cell-fate reprogramming process and the role of NEUROD1 in it remains yet to be fully understood. Further research is necessary to define the most effective conditions to achieve desired cell types, such as the most effective combination of reprogramming factors, the most amenable cell types (somatic cells, induced pluripotent stem cells (iPSC) or embryonic stem cells (ESC), and *in vivo* or *in vitro* applications using gene therapy vectors.

### 1.5 NEUROD1-driven cell-fate determining mechanisms

To elucidate the current knowledge of NEUROD1-driven cell fate acquisition, we revisited several recent studies, namely, in ESCs (Pataskar et al., 2016; Singh et al., 2022), iPSC (Choi et al., 2020; Zhang et al., 2018), mature microglia (Matsuda et al., 2019), and developing pancreatic endocrine cells (Bohuslavova et al., 2023), which inspected the molecular mechanisms behind this phenomenon.

**FIGURE 2:**
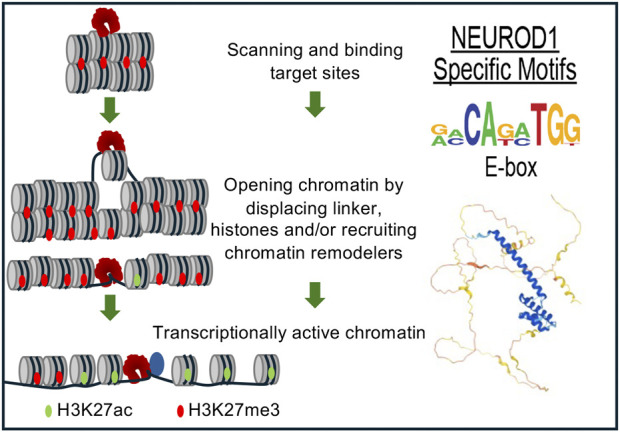
NEUROD1 reprograms chromatin to induce the neuronal program. NEUROD1 targets its target sites in heterochromatin, followed by the replacement of inactive H3K27me3 by active H3K27ac histone mark and increased chromatin accessibility, resulting in the induction of neuronal fate program (Pataskar et al., 2016).

In murine ESCs, ectopic NEUROD1 expression initiated a 48-h neurogenetic differentiation process *in vitro* accompanied by massive gene expression changes. This included the downregulation of pluripotent markers, such as OCT4, NANOG, and KLF4 (but not SOX2), and the sustained upregulation of neuronal markers such as Tuj1 (Pataskar et al., 2016). Importantly, this differentiation process occurred despite the presence of pluripotency-promoting growth factors, such as leukemia inhibitory factor (LIF), in the culture medium, without the need for additional external neuronal development signals, demonstrating a strong effect of NEUROD1 in driving cell fate. NEUROD1 promoted epigenetic changes at its target sites, initiating neuronal differentiation by inducing the loss of the repressive mark H3K27me3 and the acquisition of the active chromatin mark H3K27ac (Pataskar et al., 2016). NEUROD1 binding correlated with chromatin remodeling and increased promoter accessibility. NEUROD1 bound its target promoter sites despite their heterochromatic state and triggered their remodeling to euchromatin, as marked by increased levels of H3K27ac. Additionally, NEUROD1 displaced TBX3 from its target sites to induce neuronal gene expression, counteracting the role of TBX3 in promoting mesoendoderm lineage and suppressing neuronal fate. Similarly, NEUROD1 displaced UTF1, a key factor in maintaining the pluripotent state. Similar results were obtained *in vivo* by a short transient event of NEUROD1-expression, which induced a long-term neurogenetic effect in terms of gene expression, differentiation, and chromatin state of produced induced neuronal cells (Pataskar et al., 2016) (Figure 2). A follow-up study focused on genome-wide time-lapse propagation of the histone modification H3K27ac related to NEUROD1 binding in this process (Singh et al., 2022). While NEUROD1 initiated a significant euchromatization, only a half of the related genes was eventually activated. The selective upregulation was attributed to TCF12, which functioned as a stage-specific coregulator in the NEUROD1 expressing sub-pool of developing neurons. NEUROD1 and TCF12 physically interact with each other, and together mediate time-specific activation of several neural migratory genes. This phenomenon confirms that spatiotemporal events during differentiation are strictly regulated in a cell-type specific manner by other transcription factors and coregulators (Singh et al., 2022). Concordantly with this idea, a recent study clearly demonstrated that NEUROD1 can co-dependently operate even with other pioneering factors, namely, FOXG1 during neurogenesis (Akol et al., 2023).

Next two studies inspected the chromatin remodeling and its related accessibility change during the differentiation of human iPSC into neuronal cells (Choi et al., 2020; Zhang et al., 2018). Firstly, changes of chromatin state during induced neuronal cell differentiation are tightly linked to the binding of transcription factors, namely, NEUROD1 and NEUROG2, which are master-regulators in iPSC-to-neuronal cell induction (Zhang et al., 2018). This phenomenon was attributed to yet unknown epigenetically control mechanism. Interestingly, NEUROD1 targeted itself at the promoter region, indicating a possible self-regulation during neuronal differentiation (Zhang et al., 2018). Secondly, Choi et al. (2020) analyzed which DNA regions are modified during induced iPSC differentiation into neuronal progenitors. Using Assay for Transposase-Accessible Chromatin sequencing (ATAC-seq), they demonstrated that the extensive chromatin accessibility changes occurred predominantly in neuronal progenitor cell enhancer regions. These DNA accessibility changes corresponded with histone modification changes. Elevated levels of trimethylation of histone H3 at lysine 4 (H3K4me3, active histone marker) and H3K27ac (active histone marker) and a lower level of H3K27me3 (repressive histone marker) correlated with accessible chromatin regions of ATAC peaks in neuronal progenitor cells. NEUROD1 was identified as the main determinant in this process, initiating chromatin opening and allowing DNA access to plethora of other factors participating in the differentiation of neural progenitors (Choi et al., 2020).

Another mechanistic insight into NEUROD1-induced cell reprogramming was presented in a study on microglia-to-neuron conversion (Matsuda et al., 2019). In this study, NEUROD1 was introduced by lentiviral vector both *in vitro* into microglia and *in vivo* into the adult mouse brain. Focusing initially on DNA methylation, the study found that NEUROD1 preferentially binds to unmethylated CpG-rich regions. Despite this preference, there were no differences between upregulated neural genes and unchanged genes, indicating that this epigenetic modification affects NEUROD1 binding but not its reprogramming activity. In contrast, histone modifications significantly impacted NEUROD1 activity. The study identified an enrichment of bivalent domains—marked by both activating (H3K4me3) and repressive (H3K27me3) histone marks—at NEUROD1 binding sites of regulatory elements of target proneural genes (Matsuda et al., 2019). Bivalent domains allow for rapid gene expression changes, due to the simultaneous presence of activation and repression marks, a mechanism crucial for embryonic development (Voigt et al., 2013; Harikumar and Meshorer, 2015). Subsequently, as neuronal reprogramming progressed, these NEUROD1-bound bivalent domains transitioned to a monovalent H3K4me3 state. This transition represents a reprograming mechanism by which cells may acquire and maintain neuronal identity (Matsuda et al., 2019). Interestingly, NEUROD1 was observed to bind its own endogenous regulatory element, suggesting a potential self-regulatory loop. Moreover, NEUROD1 also bound to the promoter region of the *Jmjd3* gene, upregulating its expression. JMJD3 demethylase removes repressive H3K27me3 from bivalent domains, leading to locally opened chromatin states. An additional mechanism of NEUROD1-mediated neuronal reprograming involved the induction of the expression of transcription factors such as SCRT1 and MEIS2, which repress the microglial program, helping to facilitate microglia-to-neuron conversion. Overall, NEUROD1 selectively targeted proneural genes and activated their expression through chromatin remodeling, resulting in transdifferentiation into neuron-like cells (Matsuda et al., 2019). This discovery was subjected to an intense debate and series of follow-up studies (Matsuda-Ito et al., 2022; Irie et al., 2023; Rao and Peng, 2022; Rao et al., 2021), while eventually the results included lineage tracing and demonstrated that NEUROD1 can reprogram isolated murine microglia *in vitro*.

A recent study using the mouse model of *Neurod1* deletion demonstrated the altered chromatin landscape in the developing endocrine pancreas (Bohuslavova et al., 2023). *Neurod1* gene deficiency changed H3K27me3 profile in bivalent domains of promoters of genes essential for endocrine development. Notably, identified peaks were in the proximity of NEUROD1-binding sites. The deletion of the *Neurod1* gene disrupted the gene regulatory network and chromatin landscape, eventually compromising endocrine differentiation and the molecular identity of endocrine cells in the mouse model (Bohuslavova et al., 2023). Additionally, an enrichment of open chromatin in the enhancer regions of the *NEUROD1* gene and in regions regulated by NEUROD1 was identified in human pancreatic islets from diabetic donors compared non-diabetic controls (Bysani et al., 2019). Correspondingly, the expression levels of the *NEUROD1* mRNA were increased in diabetic islets of donors (Bysani et al., 2019). These findings suggest that epigenetic mechanisms regulate the *NEUROD1* gene expression and its ability to bind to target genes in human diabetic islets, aiming to improve insufficient insulin secretion, a hallmark of diabetes. These conclusions raise the question of whether NEUROD1 itself, alone or in coordination with other factors, directly affects the chromatin structure of diabetic islets, or if changes in the open chromatin landscape associated with NEUROD1 expression and function are indirect.

### 1.6 NEUROD1 role in neuroendocrine tumorigenesis

Understanding cell-fate determination is important not only for development but also for cancer research, influencing both the etiology and treatment of cancer. Neuroendocrine neoplasms represent a heterogenous group of tumors originating from widely distributed cells of the neuroendocrine system, expressing neuronal differentiation markers, and presenting a broad spectrum of symptoms based on the secreted peptide hormones (Bertrand et al., 2002). These tumors can emerge either *de novo* or because of therapeutic pressure (Oser et al., 2015). The primary tumors most frequently occur in the lungs (bronchial carcinoids), intestine, prostate, and pancreas. Characteristics and therapeutic management of neuroendocrine tumors largely depend on the location of the primary tumor and the degree of differentiation and dissemination. Several studies have reported that upregulation of NEUROD1 contributes to the malignant progression of neuroendocrine tumors in the prostate (Cejas et al., 2021), lungs (Huang et al., 2018; Ireland et al., 2020; Llabata et al., 2021; Pongor et al., 2022), and the brain (Lewis et al., 2022). For instance, NEUROD1 promotes tumor cell survival and metastasis in aggressive neuroendocrine lung and prostate tumors by facilitating the transformation of epithelial cells to neuronal-like cells (Cindolo et al., 2007; Pongor et al., 2022). Increased NEUROD1 expression in patient-derived xenograft (PDX) models and human clinical samples correlates with enhanced migration of neuroendocrine small cell lung and prostate cancer cells (Ikematsu et al., 2020; Cejas et al., 2021). In a mouse model, upregulation of NEUROD1 led to the differentiation of neuroendocrine medulloblastoma cells, resulting in reduced proliferation and decreased stemness and tumorigenic potential of these cells (Cheng et al., 2020). This aligns with NEUROD1’s developmental role in differentiating cerebellar granule neuron precursors, where its deletion disrupts differentiation and prolongs precursor cell proliferation (Miyata et al., 1999). Furthermore, overexpression of NEUROD1 in non-endocrine lung cancer cell lines activates a neuroendocrine program, underscoring NEUROD1’s role in inducing a neuroendocrine phenotype (Neptune et al., 2008). Differentiation therapy, which reprograms tumor cells to differentiate, thereby limiting their proliferation and subsequent tumor growth, presents a promising, less toxic, and more targeted approach to cancer treatment. This therapy can be achieved through various combinations of transcription factors, microRNAs, and alternations in the epigenetic landscapes (Gong et al., 2019; Chakraborty et al., 2023). Recent molecular analyses of clinical tissue samples demonstrated that NEUROD1 drives epigenetic reprogramming, leading to genetically and epigenetically diverse sub-populations within the same tumor (Cejas et al., 2021). The intra- and inter-patient heterogeneity presents significant challenges for effective clinical treatment, highlighting the necessity for novel therapeutic strategies. As understanding of the epigenetic basis of neuroendocrine tumors grows, therapies targeting these mechanisms are under investigation (Davies et al., 2020; Barazeghi et al., 2021). The epigenetic drugs currently approved for cancer therapy are targeting i) DNA methylation by DNA methyltransferases inhibitors and ii) histone acetylation by histone deacetylases inhibitors (Yang et al., 2010; Graca et al., 2016; Ma et al., 2020). While directly targeting transcription factors like NEUROD1, which mediate transcriptional and epigenetic changes, remains challenging, an interesting study showed that a small molecular inhibitor targeting the transcription factor ONECUT2—whose expression is linked to poor clinical outcomes—effectively reduced tumor volume and proliferation in a mouse model (Rotinen et al., 2018). Designing effective combinatorial therapeutic strategies will require deeper insights into neuroendocrine tumor biology and a better understanding of individual patient phenotypes to target key factors in specific tumor subpopulations.

## 2 Conclusion and future perspectives

We have reviewed only a part of the recent studies demonstrating the involvement of NEUROD1 in embryonic neuronal and endocrine development as well as in pathological cancer progression. NEUROD1 is able to mediate extensive reorganization of chromatin modifications (H3K4me1, H3K4me3, H3K27ac, H3K27me3) at its target sites, which are often located at enhancers and promoters, across a cohort of cell types in various stages of differentiation. These changes result in the forming of euchromatin and subsequently alter the expression of genes targeted by NEUROD1. Moreover, NEUROD1 may putatively establish self-propagating loop directly or together with other chromatin modifiers. Besides gene activation, differentiation process is also accompanied by gene downregulation, e.g., stemness genes. Based on the current state of knowledge, no study provided evidence that NEUROD1 would bind to cis-regulatory elements of genes that are switched off during the process. Hence, it is more likely that NEUROD1 may affect those genes indirectly. NEUROD1 is a potent pioneering factor capable of chromatin remodeling initiation, cooperation with other distinctly involved factors, and launch of impactful upregulation in gene expression leading to specific cell-fate commitment, neuronal and endocrine cell phenotypes. The ability of NEUROD1 to drive cell-fate commitment makes it a compelling target for therapeutical cell-programming both *in situ* and *in vitro*. Our review underscores the critical roles of timing and cell context in determining cell fate outcomes. The potential clinical applications of NEUROD1-based therapies will depend on continued research in several key areas: understanding the cell-type-specific mechanisms of NEUROD1 regulatory activity; characterizing the target genes and epigenetic modification governed by NEUROD1 in specific cell types; and developing technologies for efficient, cell-type-specific targeting of DNA- and small-molecule-based therapeutics.
